# Serum neurofilament heavy chains as early marker of motor neuron degeneration

**DOI:** 10.1002/acn3.50890

**Published:** 2019-09-13

**Authors:** Maxim De Schaepdryver, Janne Goossens, Steffi De Meyer, Andreas Jeromin, Pegah Masrori, Britta Brix, Kristl G. Claeys, Jolien Schaeverbeke, Katarzyna Adamczuk, Rik Vandenberghe, Philip Van Damme, Koen Poesen

**Affiliations:** ^1^ Laboratory for Molecular Neurobiomarker Research Department of Neurosciences KU Leuven Leuven Belgium; ^2^ Iron Horse Diagnostics, Inc Scottsdale Arizona; ^3^ Laboratory of Neurobiology Center for Brain & Disease Research VIB Leuven Belgium; ^4^ Euroimmun AG Lübeck Germany; ^5^ Laboratory for Muscle diseases and Neuropathies Department of Neurosciences KU Leuven Leuven Belgium; ^6^ Department of Neurology University Hospitals Leuven Leuven Belgium; ^7^ Laboratory for Cognitive Neurology Department of Neurosciences KU Leuven Leuven Belgium; ^8^ Experimental Neurology Department of Neurosciences KU Leuven Leuven Belgium; ^9^ Laboratory Medicine University Hospitals Leuven Leuven Belgium

## Abstract

**Objective:**

To determine whether serum phosphorylated neurofilament heavy chain (pNfH) levels are elevated before patients were diagnosed with sporadic or familial ALS, and what the prognostic value of these prediagnostic pNfH levels is.

**Methods:**

pNfH was measured via ELISA in leftovers of serum drawn for routine purposes before the time of diagnosis. These prediagnostic samples were retrieved from the biobank of the University Hospitals Leuven for 95 patients who in follow‐up received a diagnosis of ALS. Additionally, 35 patients with mild cognitive impairment (MCI) and 85 healthy controls (HC) were included in this retrospective study.

**Results:**

The median disease duration (range) from onset to prediagnostic sampling and from onset to diagnosis was 6.5 (−71.9–36.1) and 9.9 (2.0–40.7) months, respectively. Fifty‐eight percent of the prediagnostic samples had serum pNfH levels above the 95th percentile of pNfH levels measured in HC. Serum pNfH levels (median (range)) were elevated up to 18 months before the diagnosis of ALS (91 pg/mL (6–342 pg/mL)) in comparison with HC (30 pg/mL (6–146 pg/mL); *P* = 0.05), and increased during the prediagnostic stage, which was not observed in patients with MCI. Furthermore, prediagnostic pNfH levels were a univariate predictor of survival in ALS (hazard ratio (95% CI): 2.16 (1.20–3.87); *P* = 0.01).

**Interpretation:**

Our findings demonstrate that serum pNfH is elevated well before the time of diagnosis in mainly sporadic ALS patients. These results encourage to prospectively explore if pNfH has an added value to shorten the diagnostic delay in ALS.

## Introduction

Amyotrophic lateral sclerosis (ALS) is characterized by progressive degeneration of upper and lower motor neurons.^1^ To date, there is no cure for ALS. Recent studies demonstrated that the underlying ALS pathophysiology already occurs before the onset of definite symptoms related to ALS. To investigate the presymptomatic phase,^2^ researchers mainly focused on individuals bearing a genetic risk for ALS.^3^ The most commonly affected genes are *C9orf72, SOD1, TARDBP* and *FUS*, representing 10% of all cases with ALS.^4^ During the presymptomatic phase of *SOD1* mutation carriers, an increase in cortical excitability measured by transcranial magnetic resonance and a loss of lower motor neurons assessed by motor unit number estimation was discovered.^5^, ^6^ Interestingly, the latter finding was confirmed in a prospectively collected cohort of patients with ALS.^7^ Furthermore, morphometric changes in the brain of presymptomatic *C9orf72* mutation carriers have been described in comparison to noncarriers of the same family and healthy controls.^8^, ^9^

However, the diagnostic delay is around 12 months. This period, between the first symptoms related to ALS and a final diagnosis, is less studied. As the pathophysiology is initiated before the onset of symptoms, biomarkers might already by altered in the period from onset to diagnosis. Therefore, biomarkers could be instrumental to further characterize this prediagnostic period, and could potentially have a positive effect on the diagnostic delay.

Neurofilaments have been introduced as candidate diagnostic biomarkers for ALS.^10^, ^11^, ^12^, ^13^, ^14^ Elevated phosphorylated neurofilament heavy chain (pNfH) levels were, opposed to the neurofilament light chain (NfL), more specific at time of diagnosis to discriminate ALS from other diseases that at onset showed clinical symptoms reminiscent of ALS.^11^A cross‐sectional study demonstrated that both pNfH in cerebrospinal fluid (CSF) and NfL in blood or CSF were not elevated in asymptomatic familial ALS cases.^15^ However, a longitudinal study showed increased levels of serum NfL one year before symptom onset.^16^ NfL is therefore a promising and sensitive marker to predict disease onset in familial ALS. Nevertheless, a more specific marker is required to rule out other diseases and so to facilitate an earlier diagnosis of sporadic and familial ALS. Until now, it is not clear what the significance is of pNfH as a marker near the onset of disease in ALS. In this study, we evaluated pNfH levels in blood collected well before an established diagnosis of primarily sporadic ALS.

## Material & Methods

In this single center study, 95 patients obtained a diagnosis of ALS at the neuromuscular reference center of the University Hospitals Leuven according to the revised El Escorial criteria between 2007 and 2018. Serum samples, drawn for routine purposes prior to the diagnosis of ALS and stored at −20°C, were retrospectively retrieved from the biobank at the department of Laboratory Medicine of the University Hospitals Leuven. Clinical information was retrospectively obtained from the medical report closest to sampling. Muscle weakness, muscle atrophy and fasciculations were seen as evidence of lower motor neuron degeneration. Spastic tone, hyperreflexia, Hoffmann‐Trömner, Babinski, clonus, pseudobulbar, bulbar and frontal release signs (i.e. snout, root, palmomental and jaw jerk reflexes) were seen as evidence of upper motor neuron degeneration. Other clinical symptoms that were assessed include sensory disturbances, cerebellar signs (i.e. ataxia or eye movement disorder) and extrapyramidal signs. For 11 patients with ALS longitudinal prediagnostic serum samples were available, with at least 2 months between the different time points of sampling. If such serial samples were available, the sample closest to onset of the disease was selected for the prediagnostic group analysis. Serial serum samples were available for 35 patients with mild cognitive impairment (MCI), which were prospectively collected with at least 12 months in between. Furthermore, 85 healthy controls (HC) were cross‐sectionally selected from a larger community–recruited study cohort. Volunteers were recruited between 55 and 80 years of age through advertisement in local newspapers and through websites for seniors asking for participation in a scientific study at the University Hospitals Leuven. A total of 785 volunteers were screened and underwent a detailed interview about medical history, a Mini‐Mental State Examination (MMSE), general physical and neurological examination, and blood sampling.^17^ Volunteers selected for further analysis in this larger community–recruited cohort were excluded from this study. Other exclusion criteria for the HC used in this study were a MMSE < 27, a neurological or psychiatric history, a history of cancer within 5 years and a self–reported family history of a neurological disease.

A European Conformity–marked ELISA was used to quantify the serum pNfH level in duplicate (EQ 6562–9601, Euroimmun AG, Lübeck, Germany), as previously described.^10^ Serum pNfH levels below the analytical sensitivity were attributed the value of 6 pg/mL corresponding to the analytical sensitivity. The intra‐assay variability, expressed as a coefficient of variances, for two quality controls was 8.9% (36.4 pg/mL) and 5.1% (97.2 pg/mL), whereas the inter‐assay variability was 14.4% (36.4 pg/mL) and 10.2% (97.2 pg/mL).

### Standard protocol approvals, registrations, and patient consents

The retrospective study was approved by the University Hospitals Leuven Institutional Review Board (S60768). The healthy controls (S51125) and MCI patients (S55892) were collected in the framework of previous studies that were approved by the University Hospitals Leuven Institutional Review Board.

### Statistical analysis

Normality of the data was assessed with the Shapiro‐Wilk test. As transformation of the data did not result in a normal distribution, the Mann‐Whitney U test, the Kruskal‐Wallis test, corrected for multiple comparisons (Dunn's post hoc test) and the Wilcoxon signed‐rank test were used. Gender differences between cohorts were analyzed with the Chi‐squared test. Age and gender were used as covariates, when having a significant effect on the performance of serum pNfH in a logistic regression analysis. The area under the curve (AUC) constructed with the predicted probabilities of the logistic regression analysis and corresponding performance characteristics were reported if the AUC differed significantly from the AUC constructed with the pNfH values only. The sensitivity, specificity, positive likelihood ratio and the AUC with corresponding 95% CI were calculated with receiver operating characteristic (ROC) curves using MedCalc Statistical Software (MedCalc Software bvba, Ostend, Belgium). The optimal cutoff corresponded to the highest Youden Index. For individual serial measurements of pNfH in serum, an area was calculated under the curve constructed by the serial pNfH values (in pg/mL) plotted as a function of time (in months) with the first pNfH value as baseline value. This AUC_serialpNfH_ takes into account the change of serum pNfH over time on the one hand and the time period between different longitudinal samples on the other hand. The Cox proportional hazards regression analysis was performed to estimate survival (MedCalc Statistical Software, MedCalc Software bvba, Ostend, Belgium). The time to event was defined as the time between the prediagnostic sampling and death. Serum pNfH levels were stratified as low (<74 pg/mL), mid and high tertiles (> 226 pg/mL). Along with these biomarker tertiles, age at onset and bulbar sings at time of sampling, two predictors of the ALS prediction model,^18^ were entered into a multivariate Cox proportional hazards regression analysis. For 24 patients the date of the survival analysis was used for censoring the data when the patient was still alive. All statistical tests were performed at a 5% significance level.

## Results

### Characterization of patients and prediagnostic samples

The clinical departments where the prediagnostic samples were sampled are listed in Table 1. The differential diagnosis at time of sampling is listed in Table S1 for every individual. Most of the prediagnostic samples (*n* = 75 [79.0%]) were collected at a neurology department. At the time of sampling 69 (72.6%) patients had a differential diagnosis including a motor neuron disease. Detailed clinical information of the prediagnostic patients is provided in Table 2. A total of 63 (60%) prediagnostic patients had both signs of upper and lower motor neuron degeneration. While, signs of upper or lower motor neuron degeneration only were observed in seven (7%) and 15 (14%) patients, respectively. The median time from onset to sampling (6.5 months, range: −71.9 to 36.1) was significantly smaller in comparison with the disease duration from onset to the diagnosis of ALS (9.9 months, range: 2.0–40.7; *P* < 0.001). At the time of diagnosis, patients were classified according to the revised El Escorial criteria: suspected ALS (*n* = 7 [7.4%]), possible ALS (*n* = 19 [20%]), probable ALS (*n* = 27 [28.4%]), probable ALS lab supported (*n* = 27 [28.4%]) and definite ALS (*n* = 15 [15.8%]). No significant differences were seen in terms of serum pNfH levels between the diagnostic groups. A significant difference in age was found between the ALS cohort at time of prediagnostic sampling and the healthy controls (HC; *P* < 0.001; Table 1). The gender distribution was not significantly different between the ALS cohort and the HC *(P = *0.347; Table 1).

**Table 1:** Characteristics of the prediagnostic ALS samples, MCI and healthy controls.

	Prediagnostic ALS samples	MCI	Healthy controls
*n* (m/f)	95 (58/37)	35 (19/16)	85 (46/39)
Age at sampling	63 (26–80)	70 (55–83)	68 (57–80)
Serum pNfH (pg/mL)	154 (6–1891)	52 (6–234)	30 (6–147)
Origin sample (*n*):			
Neurology	75		
Internal medicine	11		
Hematology	3		
Cardiology	2		
Intensive care	1		
Organ transplantation	1		
Traumatology	1		
Orthopedics	1		

Median and range are given.

ALS, amyotrophic lateral sclerosis, MCI, mild cognitive impairment, pNfH, phosphorylated neurofilament heavy chain.

**Table 2:** Clinical characterization of the prediagnostic ALS cohort.

Clinical signs of	Patients tested (*n*)	Positive (*n*)	Serum pNfH (pg/mL)
Lower motor neuron degeneration			
Muscle weakness	87	75	162 (6–1633)
Muscle atrophy	86	52	163 (6–1633)
Fasciculations	85	44	201 (19–905)
Upper motor neuron degeneration			
Spastic tone	85	32	164 (7–905)
Hyperreflexia	86	54	151 (6–1633)
Hoffmann's reflex	72	34	155 (6–905)
Babinski	83	20	216 (7–905)
Clonus	79	14	176 (47–905)
Pseudobulbar	87	12	202 (6–436)
Bulbar	86	28	201 (25–436)
Frontal release signs	64	39	152 (6–905)
Other			
Sensory disturbances	81	31	134 (6–1891)
Cerebellar signs	82	15	162 (6–1891)
Extrapyramidal signs	12	4	173 (145–312)

Median serum pNfH and range are given for the positive cases. Frontal release signs: snout, root, palmomental or jaw jerk reflexes. Cerebellar signs: ataxia or eye movement disorder. pNfH: phosphorylated neurofilament heavy chain.

### Serum pNfH levels before the diagnosis of ALS

**Figure 1:**
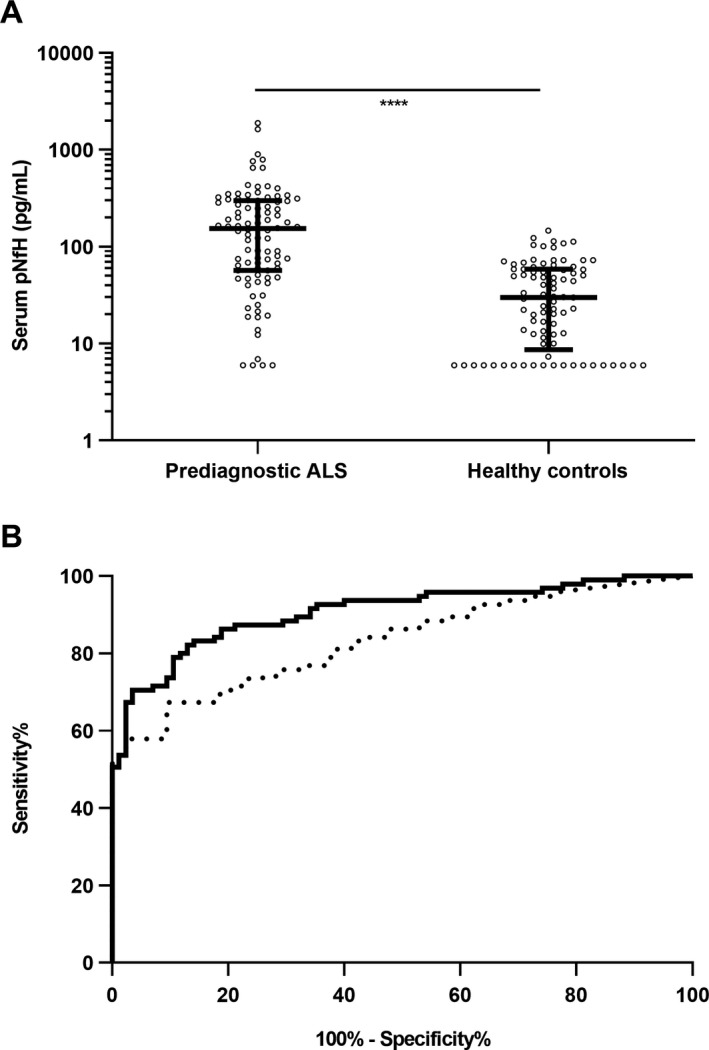
Serum pNfH levels at a prediagnostic ALS stage in comparison with healthy controls. (A) Scatter plot showing phosphorylated neurofilament heavy chain (pNfH) levels in serum of samples collected before the diagnosis of amyotrophic lateral sclerosis (prediagnostic ALS) and healthy controls (HC). Median and interquartile range are presented on top of the plot. Mann‐Whitney U test (****P < 0.0001). (B) Receiver operating characteristic curve to discriminate prediagnostic ALS from HC based upon serum pNfH levels (dotted line) and adjusted for age (solid line).

The median serum pNfH levels were significantly higher in prediagnostic samples of patients with ALS in comparison to HC *(P < *0.001; Fig. 1A; Table 1). Genetic analysis of *C9orf72, SOD1, TARDBP* and *FUS* was available for 61 (64,2%) patients with ALS and revealed 15 mutation carriers (Table S1). Prediagnostic serum pNfH levels were not significantly different between sporadic and familial ALS (*P* = 0.128). A significant positive correlation between prediagnostic serum pNfH levels and age at blood sampling was found in the ALS cohort (*r*_s_ = 0.29, CI: 0.08–0.47; *P* = 0.005), but not in the HC cohort *(P = *0.570). In line with this, logistic regression analysis including pNfH in different cohorts (prediagnostic ALS and HC) and age as covariates yielded an AUC that was significantly different (0.907, CI: 0.855–0.945) as compared to the AUC in a ROC analysis including pNfH as a single variable (0.832, CI: 0.769–0.883) *(P* = 0.003; Fig. 1B). The optimal cutoff, determined by ROC analysis, was 73.4 pg/mL. This cutoff yielded a sensitivity of 67.4% (CI: 57.0–76.6%), a specificity of 90.6% (CI: 82.3 – 95.8%) and a positive likelihood ratio of 7.2 (CI: 3.6–14.0). The optimal cutoff, determined by ROC analysis using the age adjusted predicted probabilities from the logistic regression, corresponded with a sensitivity of 82.1% (CI: 72.9–89.2%), a specificity of 87.1% (CI: 78.0–93.4%). The positive likelihood ratio was 6.3 (CI: 3.6–11.1).

**Figure 2:**
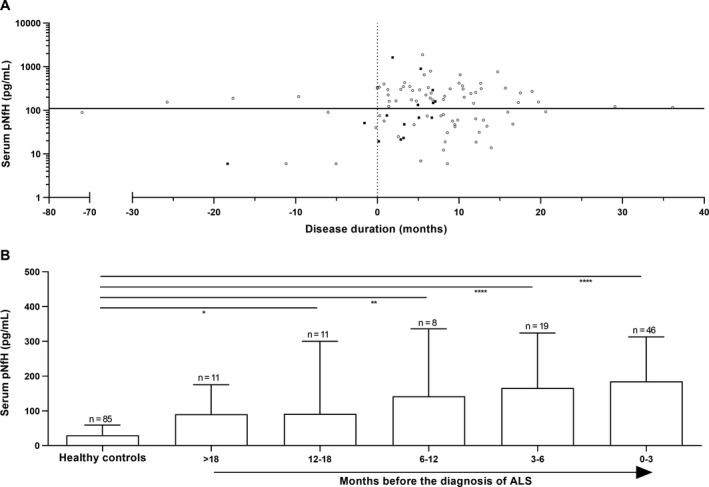
Prediagnostic serum pNfH levels as function of disease duration and months before diagnosis of ALS. (A) Scatter plot showing individual phosphorylated neurofilament heavy chain (pNfH) levels in serum as a function of the disease duration. The horizontal solid line represents the 95th percentile of serum pNfH levels calculated in a healthy cohort. Ten samples were collected before the time of the disease onset (vertical dotted line). Black rectangles represent the patients with familial ALS (*n* = 15) (B) The histogram shows pNfH levels in serum as function of months before the diagnosis of amyotrophic lateral sclerosis (ALS) is obtained. The median and interquartile range are given. Kruskal‐Wallis test, corrected for multiple comparisons (Dunn's post hoc test, ****P < 0.0001, **P < 0.01, *P < 0.05), to compare the prediagnostic ALS groups with the healthy controls.

The 95th percentile of the pNfH levels from the HC was equal to 111 pg/mL. When applying this cutoff to all individual prediagnostic ALS samples as a function of the disease duration (Fig. 2A), already 57.9% of the prediagnostic samples had a serum pNfH level above this threshold as early as 26 months before the onset of symptoms. Next, we classified the patients according to the time of sampling before the diagnosis of ALS. Serum pNfH levels increased towards diagnosis and were significantly elevated up to 18 months before the diagnosis of ALS (median: 91 pg/mL, range: 6–342 pg/mL) in comparison with HC (median: 30 pg/mL, range: 6–146 pg/mL; *P* = 0.05; Fig. 2B).

**Figure 3:**
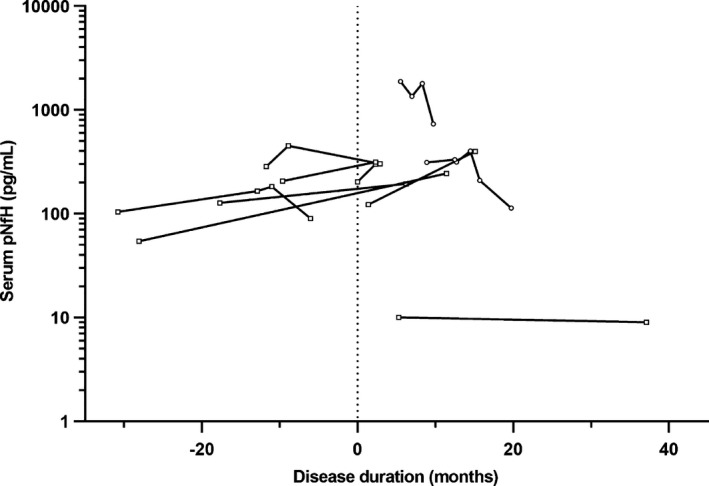
Longitudinal prediagnostic serum pNfH levels in patients with ALS. Eleven patients with amyotrophic lateral sclerosis (ALS) had multiple longitudinal prediagnostic samples with a time difference of at least 2 months. A subset of patients (*n* = 8) with an initial serum phosphorylated neurofilament heavy chain (pNfH) below 300 pg/mL is highlighted with rectangle symbols. The vertical dotted line represents the disease onset, data preceding this line mark the presymptomatic stage.

**Figure 4:**
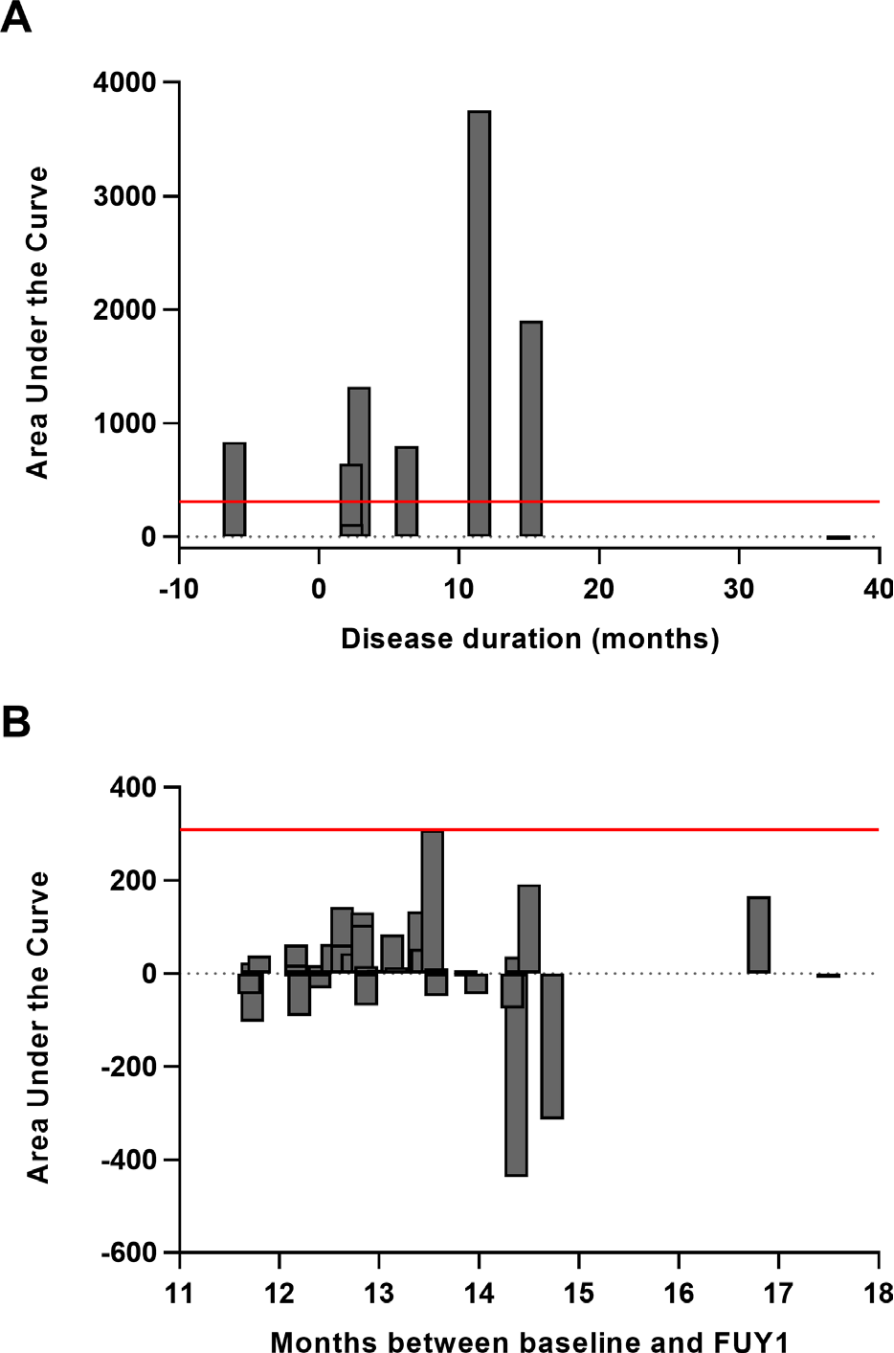
The change of serum pNfH levels over time in prediagnostic samples of patients with ALS. (A) The area under the curve (AUC_serialpNfH_) was calculated for eight patients with a prediagnostic serum pNfH concentration below 300 pg/mL and serial measurement(s) at least 2 months after sampling of the former prediagnostic serum. For each patient the AUC_serialpNfH_ was plotted as a function of the last time point of serial sampling (B) The change of serum pNfH from baseline to follow‐up year 1 (FUY1) was quantified by calculating the AUC_serialpNfH_ for 35 patients with mild cognitive impairment (MCI). The vertical solid line corresponds to the highest change of the AUC_serialpNfH_ observed in MCI patients. Notice that figure A and B are not equally scaled on the Y‐axis.

The individual changes of serum pNfH during the prediagnostic stage are shown in Figure 3. In eight patients with an initial serum pNfH level below 300 pg/mL, a clear increase of serum pNfH (median AUC_serialpNfH_: 819, range: −16 to 3,755) over a median time period of 19 months (range: 2 – 40 months) was seen (Fig. 4 A). This rise of pNfH may be specific to motor neuron degeneration as patients with MCI have on average a significantly smaller increase over a one year period (median AUC_serialpNfH_: 18, range: −437 to 309; *P* < 0.001; Fig. 4B).

### Serum pNfH levels at the presymptomatic stage

Ten (10.5%) prediagnostic samples were collected before the first symptoms related to ALS (Fig. 2A, Table S1). Three patients had a serum pNfH level above the aforementioned cutoff of 111 pg/mL as early as 26 months before the onset of symptoms related to ALS. Clinical data of all ten patients with a presymptomatic sample were collected at diagnosis and are listed in Table 3. The time from sampling to symptom onset and the survival after diagnosis did not differ between the patients with high (>111 pg/mL) serum pNfH level compared to those with a low (<111 pg/mL) serum pNfH level.

**Table 3:** Clinical data of patients with ALS with a presymptomatic sample

Serum pNfH level	Sampling to symptom onset (months)	Survival after diagnosis (months)	Regions with LMN signs^1^	Regions with UMN signs^1^	Site of onset^1^
>111 pg/mL^2^					
Patient 1	9,6	9,1	Bulbar, UL, LL	Bulbar, UL, LL	Spinal
Patient 2	17,7	6,7	None	UL, LL	Spinal
Patient 3	25,7	2,8	UL, LL	Bulbar, UL, LL	Generalized
<111 pg/mL^2^					
Patient 4	6,0	12,0	Bulbar, UL	Bulbar, UL	Bulbar
Patient 5	71,9	6,9	UL	Bulbar, UL, LL	Spinal
Patient 6	1,6	18,8	LL	Bulbar, UL, LL	Spinal
Patient 7	0,2	4,7	Bulbar	None	Thoracic/Respiratory
Patient 8	5,1	11,5	UL	Bulbar, UL	Spinal
Patient 9	11,2	3,1	UL, LL	Bulbar, UL, LL	Spinal
Patient 10	18,3	5,7	UL	Bulbar, UL, LL	Bulbar

1 Lower motor neuron (LMN), upper motor neuron (UMN) signs and site of onset were assessed by clinical assessment at the time of diagnosis.

2 Cutoff defined as the 95th percentile of the serum phosphorylated neurofilament heavy chain (pNfH) levels from the HC. UL: upper limbs, LL: lower limbs

### Serum pNfH levels as prognostic marker for ALS

The univariate survival analysis revealed that patients with high prediagnostic serum pNfH levels had a shorter survival than patients with low prediagnostic serum pNfH levels (hazard ratio: 2.16, CI: 1.20–3.87; *P* = 0.01). When age at onset and bulbar symptoms present at time of sampling were added in a multivariate survival analysis, the prediagnostic serum pNfH levels were not any longer an independent predictor of survival (hazard ratio: 1.59, CI: 0.83–3.04*; P* = 0.161). Instead, bulbar symptoms present at time of sampling were significant predictors of survival (hazard ratio: 2.41, CI: 1.20–4.85*; P* = 0.013).

## Discussion

We characterized pNfH levels and their change in serum samples collected before patients were diagnosed with ALS. Moreover, we defined the normal serum pNfH range in a large cohort of healthy controls. In the prediagnostic stage of ALS, a significant increase of serum pNfH levels was found up to 18 months before the diagnosis. The levels further increased towards diagnosis. Fifty‐eight percent of the prediagnostic ALS samples had serum pNfH levels higher than the 95th percentile pNfH value of the HC cohort. Strikingly, one sporadic patient had a serum pNfH level above this threshold 26 months before the onset of the symptoms related to ALS. When we applied our previously defined cutoff of 81.9 pg/mL to differentiate ALS mimics from patients with ALS at time of diagnosis,^10^ 62% of the prediagnostic ALS samples had serum pNfH levels above this cutoff. Interestingly, diseases like multifocal motor neuropathies, radiculopathies and cervical stenosis that were used as ALS mimics in our previous study to determine the cutoff were also observed in the differential diagnoses of the prediagnostic sample cohort in this study. From the ten presymptomatic patients included in this study, already five patients had a serum pNfH level above the aforementioned cutoff of 81.9 pg/mL.

These results were in accordance with a recent study on the neurofilament light chain (NfL), which included presymptomatic patients at genetic risk for ALS, demonstrating that serum NfL levels were elevated up to 12 months before symptom onset.^16^ In contrast, it has been reported that asymptomatic mutation carriers had normal serum NfL levels that were not different from non‐mutation carrier controls.^15^ However, it should be noticed that in the latter study most of samples of the asymptomatic mutation carriers were collected more than 24 months before the onset of symptoms. Most studies investigating levels of neurofilaments before the diagnosis focused on patients at genetic risk for ALS and on the NfL. An important advantage in our study is that we mainly retrieved prediagnostic samples of patients with sporadic ALS, and that we evaluated pNfH, which has been shown to be a more specific diagnostic biomarker in ALS than NfL.^11^ Supporting our findings is the fact that in patients with sporadic ALS diagnosed early, pNfH levels were already significantly elevated in CSF and blood.^10^, ^19^, ^20^

The cutoff determined via ROC analysis using the predicted probabilities of logistic regression, resulted in a specificity and positive predictive value of 87% and 82%, respectively, with a positive likelihood of 6.3. This is comparable with previous studies reporting performance characteristics of neurofilaments at an early ALS disease stage.^19^, ^20^ Nevertheless, this study indicates that a selection bias towards age might significantly influence the cutoff determined via ROC analysis. Indeed, our results show a significant effect of age on the area under the curve determined by logistic regression for pNfH differentiating between prediagnostic ALS samples and HC. This effect is in accordance with the significant difference of age between our patients with ALS and the HC. In addition, the effect could be explained by the inclusion of younger patients who later on developed ALS, whose pNfH levels were normal in the prediagnostic serum sample. These findings are supported by the longitudinal data that show an increase of pNfH levels over time in prediagnostic samples. This increase was specifically observed in those cases whose levels of serum pNfH were below 300 pg/mL in the first prediagnostic sample retrieved. Remarkably, the extent of the increase seemed to be related to motor neuron degeneration as the magnitude of the rise of serum pNfH was lower in patients with MCI. The rise of serum pNfH in the early prediagnostic stage of ALS is in line with findings of others demonstrating that NfL just started to rise in blood of patients with familial ALS in the close proximity of the onset of the disease.^16^

Finally, we investigated the prognostic value of our prediagnostic serum pNfH levels to predict the survival of the patient with ALS. A twofold increased risk of death was found when having a serum pNfH level above 226 pg/mL. Next, we investigated what parameters from the ALS prediction model we could incorporate in our survival analysis based upon the available data the clinician had at time of sampling and decided to include age and bulbar onset.^18^ The results of the multivariate survival analysis highlighted that serum pNfH levels were no longer a predictor of survival, but instead, the presence of bulbar symptoms became a more powerful predictor of survival. Nonetheless, bulbar symptoms were only seen in 17% of the prediagnostic ALS patients, meaning that for 83% of the prediagnostic serum pNfH levels remain a good predictive biomarker to estimate survival.

In conclusion, we demonstrated that serum pNfH levels are increased in patients with ALS before they obtain their final diagnosis. This finding was in accordance with presymptomatic findings on serum NfL. Furthermore, in the absence of bulbar signs, prediagnostic serum pNfH levels in the highest tertile were associated with a higher risk of death, highlighting the prognostic added value of the biomarker. These results encourage us to further investigate the serum pNfH levels at a prediagnostic stage and if they might be able to decrease the diagnostic delay in patients with ALS.

## Author Contribution

M.D.S., A.J., P.V.D., and K.P. developed the concept and design of the study. M.D.S., A.J., B.B., P.V.D., and K.P. drafted the manuscript and figures. M.D.S., J.G., S.D.M., P.M., J.S., K.A., K.G.C., R.V., and K.P. were involved in the acquisition, analysis and interpretation of data. Critical revision of the manuscript for important intellectual content was done by all authors.

## Conflict of Interest

M.D.S., J.G., S.D.M., P.M., J.S., K.A., K.G.C., R.V., and P.V.D. have nothing to report. K.P. received ELISA kits that were sponsored by Euroimmun AG (Lübeck, Germany). B.B. receives personal fees as a full time employee of Euroimmun AG (Lübeck, Germany). A.J. is a paid employee and stock holder of Iron Horse Diagnostics, Inc. (Scottsdale, AZ, USA).

## Supporting information


**Table S1.** The differential diagnosis per patient at time of sampling.
