# Supplementary material for: Serum neurofilament heavy chains as early marker of motor neuron degeneration
Source: Ann Clin Transl Neurol. 2019 Sep 13;6(10):1971–9. doi: 10.1002/acn3.50890 (PMC6801162; doi:10.1002/acn3.50890)
Supplement: Supplementary file 1 — Table S1. The differential diagnosis per patient at time of sampling. [file ACN3-6-1971-s001.docx]

**Serum neurofilament heavy chains as early marker of motor neuron degeneration**

**Supplementary table 1**

*Authors*

Maxim De Schaepdryver, MSc.^1^; Janne Goossens, MSc.^1^; Steffi De Meyer, BSc.^1^; Andreas Jeromin, PhD^2^; Pegah Masrori, MD^3,6,8^; Britta Brix, PhD^4^; Kristl G. Claeys, MD PhD Prof^5,6^; Jolien Schaeverbeke, PhD^7^; Katarzyna Adamczuk, PhD^7^; Rik Vandenberghe, MD PhD Prof^6,7^; Philip Van Damme, MD PhD Prof^3,6,8,^*; Koen Poesen, PharmD PhD Prof^1,9,^*

**Supplementary table 1 The differential diagnosis per patient at time of sampling**

| **Patient** | **Differential diagnosis at sampling** | **Gene mutation^a^** |
| --- | --- | --- |
| 1 | Presymptomatic, familial CMT |  |
| 2 | Paraneoplastic syndrome, MSA, *Radiculopathy* |  |
| 3 | MND, Neuropathy (AIDP, CIDP or AMAN) |  |
| 4 | Presymptomatic, Acute myeloid leukemia |  |
| 5 | Presymptomatic, Cancer-related weight loss |  |
| 6 | *Myasthenia gravis*, Pomp disease, inflammatory myopathy, MND |  |
| 7 | ALS |  |
| 8 | Presymptomatic, Autoimmune adrenal disease |  |
| 9 | ALS | C9orf72 |
| 10 | MND |  |
| 11 | ALS |  |
| 12 | ALS |  |
| 13 | ALS |  |
| 14 | Spasmodic paraplegia, Adrenoleukodystrophy, PLS, *MS* |  |
| 15 | *Myasthenia gravis*, MND |  |
| 16 | ALS, Cerebral pathology |  |
| 17 | ALS |  |
| 18 | ALS |  |
| 19 | ALS | FUS |
| 20 | MND, Vascular disease, Progressive supranuclear palsy |  |
| 21 | MND |  |
| 22 | Axonal polyneuropathy |  |
| 23 | Cerebellar degeneration, Mitochondrial myopathy |  |
| 24 | ALS |  |
| 25 | ALS |  |
| 26 | Presymptomatic |  |
| 27 | Inclusion body myositis |  |
| 28 | Medullary compression |  |
| 29 | Neuromuscular junction disease, Parkinsonism, MND |  |
| 30 | ALS |  |
| 31 | ALS |  |
| 32 | ALS |  |
| 33 | MMN, ALS |  |
| 34 | ALS |  |
| 35 | CMT, dHMN, Cervical myelomalacia, MND with polyneuropathy |  |
| 36 | MND, Polyneuritis |  |
| 37 | ALS, *Myasthenia Gravis* |  |
| 38 | ALS |  |
| 39 | MND |  |
| 40 | Peroneal neuropathy, Steroid myopathy, MND |  |
| 41 | ALS |  |
| 42 | MND, Vascular disease |  |
| 43 | *Radiculopathy*, ALS |  |
| 44 | MND, FTD |  |
| 45 | Cerebral pathology |  |
| 46 | PLS, Myelopathy | C9orf72 |
| 47 | ALS |  |
| 48 | MND |  |
| 49 | MND, *Motor polyradiculopathy* |  |
| 50 | MND, *Plexopathy*, cervical myelopathy |  |
| 51 | ALS |  |
| 52 | Presymptomatic, Follow-up left ventricular hypertrophy |  |
| 53 | *Cervical radiculopathy*, ALS-FTD |  |
| 54 | ALS |  |
| 55 | *Cervical/Lumbar spinal stenosis* |  |
| 56 | Cramp-fasciculation syndrome |  |
| 57 | Spinal cord compression | SOD1 |
| 58 | Parkinson plus, MSA |  |
| 59 | ALS |  |
| 60 | Multipathology, *Cervical stenosis with myelopathy*, CIDP |  |
| 61 | MND, Polyneuropathy related to coeliac disease |  |
| 62 | ALS | C9orf72, TARDBP |
| 63 | Cervical Myelomalacia, MND, Paraneoplastic | C9orf72 |
| 64 | ALS |  |
| 65 | MND | FUS |
| 66 | MND, Sensory neuropathy | C9orf72 |
| 67 | MND, *Myasthenia gravis*, Myelomalacia |  |
| 68 | MND | FUS |
| 69 | *Plexopathy*, CIDP, MND |  |
| 70 | MMN, ALS | TARDBP |
| 71 | Presymptomatic, Follow-up familial history of ALS | SOD1 |
| 72 | ALS |  |
| 73 | ALS | C9orf72, TARDBP |
| 74 | Sensorimotor polyneuropathy |  |
| 75 | Presymptomatic, Tibia external rotation syndrome | SOD1 |
| 76 | ALS |  |
| 77 | CIDP, Stroke |  |
| 78 | ALS, Cervical myelopathy |  |
| 79 | *MMN*, ALS |  |
| 80 | Spondylodiscitis |  |
| 81 | Presymptomatic, No rheumatic disorder |  |
| 82 | Neuroborreliosis, *Cervical stenosis* |  |
| 83 | ALS |  |
| 84 | MND |  |
| 85 | ALS, *MMN* |  |
| 86 | Stroke |  |
| 87 | *Radiation-induced brachial plexopathy, MMN*, Vasculitis, MND |  |
| 88 | ALS |  |
| 89 | ALS, Space occupying lesion | C9orf72 |
| 90 | MND, Kennedy disease, *Polyradiculopathy*, *MMN*, CIDP |  |
| 91 | Viral meningitis |  |
| 92 | ALS |  |
| 93 | MND | C9orf72 |
| 94 | ALS |  |
| 95 | Presymptomatic, Kidney transplantation |  |

Differential diagnoses in italic were used as a disease mimic of patients with amyotrophic lateral sclerosis (ALS; De Schaepdryver M, et al. 2018. J Neurol Neurosurg Psychiatry). AIDP: Acute inflammatory demyelinating polyradiculoneuropathy, AMAN: Acute motor axonal neuropathy, CIDP: Chronic inflammatory demyelinating polyradiculoneuropathy, CMT: Charcot-Marie-Tooth disease, dHMN: Distal hereditary motor neuropathy, FTD: Frontotemporal lobar degeneration, MMN: Multifocal motor neuropathy, MND: Motor neuron disease, MS: Multiple sclerosis, MSA: Multiple system atrophy, PLS: Primary lateral sclerosis, PSP: Progressive supranuclear palsy. ^a^: Genetic analysis at time of diagnosis unless stated otherwise.
